# Reduced work absenteeism in patients with hepatitis C treated with second‐generation direct‐acting antivirals

**DOI:** 10.1111/jvh.13398

**Published:** 2020-09-22

**Authors:** Peter Lindgren, Sofia Löfvendahl, Gunnar Brådvik, Ola Weiland

**Affiliations:** ^1^ Department of Learning Informatics, Management and Ethics Karolinska Institutet Stockholm Sweden; ^2^ The Swedish Institute for Health Economics Lund Sweden; ^3^ Department of Medicine Division of Infectious Diseases Karolinska Institutet Karolinska University Hospital Huddinge Stockholm Sweden

**Keywords:** absenteeism, antiretroviral agents, hepatitis C, registries, sick leave

## Abstract

The cost‐effectiveness of the second‐generation direct‐acting antivirals (DAA) has received considerable attention; however, their effect on wider societal costs has remained relatively unexplored. The aim of this study was to investigate the effect the new drugs have on sick leave compared to older treatment paradigms. This retrospective study utilized Swedish registry data to identify three cohorts: (a) patients treated with ribavirin and/or peginterferons (peg‐IFN) during 2005‐2011; (b) patients treated with the first generation of DAAs and ribavirin and/or peg‐IFN 2011‐2013; and (c) patients treated with the new generation of DAAs 2014‐2018. Individual‐level data on sick leave and early retirement were used to compare days away from work the year prior to the year following treatment initiation. A difference‐in‐difference model was estimated to test for differences between the cohorts adjusting for age and gender. Days away from work prior to treatment initiation was similar in the cohorts: 106, 85 and 94 days in cohorts 1 to 3. After treatment initiation, the number of days away from worked increased in cohort one and two to 150 and 140 days, while it remained similar in cohort three (88 days). The monetary value of the avoided sick leave was 7000‐10 000 €. In conclusion, patients treated with second‐generation DAAs without peg‐IFN had fewer days of sick leave in the year following treatment initiation compared to older treatments. Some caution is advised when interpreting the absolute figures due to potential heterogeneity between cohorts as they were treated at different points in time.

## 1 INTRODUCTION

The introduction of IFN‐free treatment with second‐generation direct‐acting antiviral (DAA) drugs constituted a major step change in the treatment of patients with hepatitis C. Earlier available treatment options had offered a sustained viral response (SVR) rate in between 50% and 80% of patients depending on genotype and treatment regimen, and the current generation of DAA therapy has yielded SVR rates well above 95% (93%‐100%) depending on genotype and category of patients treated in clinical practice.^1^, ^2^

The price of the new drugs in combination with the potentially large number of eligible patients led to discussions both about the cost‐effectiveness, that is the value for money, and the affordability (or budget impact) of the new drugs. Consequently, a large number of cost‐effectiveness analyses of varying quality have been published. The vast majority of published studies consider the perspective of the healthcare payer and thus focus only on costs in the healthcare sector.^3^ A recent review of studies on the economic burden of hepatitis C infection indicates that non–healthcare costs may in fact be higher than the healthcare costs.^4^ With one exception, the reviewed studies were conducted before the second‐generation DAAs became available. There is thus a paucity of recent data on a potentially important component of the costs associated with hepatitis C treatment.

The objective of this study was to investigate the impact on work absenteeism, in this case defined as sick leave or early retirement, associated with different treatment paradigms. This was done through retrospective record linkage of patients treated for hepatitis C at different time periods in the county of Stockholm, Sweden.

## 2 MATERIALS AND METHODS

### 2.1 Data

We identified three historical cohorts of patients in the county of Stockholm receiving treatment for hepatitis C: cohort 1 initiated treatment with ribavirin and/or peginterferon (peg‐IFN) between 2005 and 2011, patients in cohort 2 were treated with telaprevir or boceprevir plus ribavirin and/or peg‐IFN between 2011 and 2013, and cohort 3 consisted of patients receiving IFN‐free treatment with any of the current (second) generation of DAAs between 2014 and 2018. Cohorts 1 and 2 were identified using the National Prescribed Drug Register with the National Patient Register being used to confirm a diagnosis of hepatitis C.^5^, ^6^ Cohort 3 was based on the national quality register for hepatitis C (InfCare Hepatitis C).^7^ To be included in the study, patients had to have filled one prescription for one of the drugs specified above and be in working ages, defined as being 19 to 67 years of age. No exclusion criteria were applied.

Utilizing their unique national identification number, treatment records were matched with data on long‐term sick leave (>14 days of absence) and early retirement from the research database at the Swedish Social Insurance Agency (Försäkringskassan).^8^ Both sick leave and disability pension can be full‐time (100%) or part‐time (eg, 25%, 50%, 75%). The number of days with part‐time compensation was adjusted to days of full compensation.

To estimate the value of foregone production to society, that is the indirect costs, we relied on the human‐capital approach valuing each day lost according to the mean salary (including employer contributions and typical pension plan contributions) for men and women, respectively.^9^ This was based on the latest available data from Statistics Sweden and Ekonomifakta.^10^, ^11^ Costs were expressed in 2018 € (1 € = 10.26 Swedish Krona).

### 2.2 Statistical methods

Using each patient as his or her own control, we calculated the difference between the number of days away from work due to sick leave or early retirement in the year prior to treatment initiation and the year following the start of treatment. To formally test for difference between the treatment groups while adjusting for population characteristics, we conducted a difference‐in‐difference regression with age and gender as covariates.^12^

### 2.3 Ethical considerations

The study was approved by the ethical review board in Stockholm (2017/2243‐31). Matching of patient data was conducted by the National Board of Health and Welfare; the researchers only had access to anonymized data during the conduct of the study.

## 3 RESULTS

A brief overview of the characteristics of the three cohorts is given in Table 1. It can be noted that there was a much smaller number of patients in cohort 2 (treated with telaprevir or boceprevir plus peg‐IFN and ribavirin) compared with the other two cohorts. This is explained by the fact that these drugs were on the market for only a short time before being replaced by the more efficient current generation of drugs and only indicated for a subset of patients in the majority with genotype 1 infection. Cohort 1 (treated with ribavirin and/or peg‐IFN) was somewhat younger and contained more women.

**TABLE 1:** Patient characteristics, days off work and value of lost production the year prior to and the year following treatment initiation

	Cohort 1			Cohort 2			Cohort 3		
Years of inclusion	2005‐2011			2011‐2013			2014‐2018		
Treatments included	Ribavirin, peg‐IFN alfa‐2a, peg‐IFN alfa‐2b			Telaprevir, boceprevir, ribavirin, peg‐IFN alfa‐2a, peg‐IFN alfa‐2b			Current generation of DAAs		
N	1511			199			2303		
Women, n (%)	583 (38.6)			65 (32.7)			805 (34.9)		
Mean age (sd)	48.5 (10.1)			52.3 (9.5)			51.0 (10.9)		
	**Year − 1**	**Year + 1**	**Diff.**	**Year − 1**	**Year + 1**	**Diff.**	**Year − 1**	**Year + 1**	**Diff.**
Any sick leave or disability pension (%)	680 (45.0)	944 (62.6)	264	81 (38.4)	121 (57.3)	49	858 (37.3)	803 (34.9)	−55
Any sick leave ≥ 14 days (%)	342 (22.6)	610 (40.4)	268	49 (23.2)	89 (42.2)	40	365 (15.8)	332 (14.4)	−33
Any disability pension (%)	385 (25.5)	386 (25.5)	1	36 (17.1)	38 (18.0)	2	517 (22.4)	489 (21.2)	−28
Mean days (SD)									
Total days	106 (150)	150 (150)	44*	85 (136)	144 (149)	59*	94 (148)	88 (143)	−6 (NS)
Days on sick leave	29 (76)	70 (110)	42*	30 (80)	90 (126)	60*	22 (70)	21 (69)	−1 (NS)
Days on disability pension	78 (142)	80 (144)	2 (NS)	55 (124)	54 (121)	−1 (NS)	72 (141)	67 (136)	−5 (NS)
Value of lost production (SD) 2018 €									
Total cost	18 035 (25 727)	25 299 (25 973)	7264*	14 577 (23 511)	24 428 (25 391)	9851*	16 103 (25 459)	15 020 (24 680)	−1083 (NS)
Cost due to sick leave	4795 (12 826)	11 643 (18 372)	6848*	5096 (13 813)	15 151 (21 239)	10 055*	3739 (11 985)	3516 (11 673)	−223 (NS)
Cost due to disability pension	13 239 (24 467)	13 656 (24 805)	417 (NS)	9481 (21 367)	9277 (20 888)	−204 (NS)	12 364 (24 206)	11 504 (23 396)	−860 (NS)

Note

1 € = 10.26 SEK (Swedish Krona).

Abbreviations: DAA, direct‐acting antiviral; Diff., difference; NS, No statistically significant difference; SD, standard deviation.

* Statistically significant difference at a level of less than .01.

In the year prior to treatment initiation, there was a somewhat larger proportion receiving either compensations for sick leave or disability pension (it is possible to receive both during the course of a year) in cohort 1 compared to the two more recent cohorts (see Table 1). A larger proportion of patients was on sick leave in cohort 2 compared to cohort 3, while a larger proportion of patients in cohort 3 received disability pension resulting in a similar number of patients receiving any benefits in the two groups. The mean number of days away from work during the year preceding treatment ranged from 85 in cohort 2 to 106 in cohort 1, with cohort 3 falling in between with 94 days.

As expected, there was no change in the number of working days lost due to early retirement in the year following treatment initiation compared to the preceding year, with only numerically small differences observed within the three cohorts. While there was a marked increase in the number of days of sick leave in cohorts 1 and 2 (42 and 60 additional days in cohorts 1 and 2, respectively), no such change could be observed among the patients treated with IFN‐free treatment with the new generation of DAA drugs. These results were confirmed in the difference‐in‐difference analysis (Table 2). In cohort 1, which was used as the reference group, patients had 41 (95% CI 35‐47) additional days of sick leave in the year after treatment initiation. Cohort 2 had 19 (95% CI 1‐36) days more, while cohort 3 had 42 (95% CI −50‐−35) days less. Adjusting for age and gender had a very limited effect on these results. Also, in line with the crude observed results, there were only small changes in days with disability pension after treatment initiation, and no difference in this change between the treatment groups.

**TABLE 2:** Results from difference‐in‐difference regressions on the number of days away from work due to sick leave (SL) or disability pension (DP) in the year following treatment initiation

Variables	(1)	(2)	(3)	(4)
	SL (unadjusted)	SL (adjusted)	DP (unadjusted)	DP (adjusted)
Year after start of treatment	41.18***	41.18***	2.42	2.42
	(35.36‐47.01)	(35.37‐47.00)	(−7.54‐12.38)	(−7.34‐12.19)
Cohort 2^a^	1.29	1.24	‐22.45**	‐32.53***
	(−10.78‐13.36)	(−10.82‐13.31)	(−43.10‐−1.81)	(−52.80‐−12.26)
Cohort 3^a^	−6.67**	−6.73**	−5.27	−11.92***
	(−11.97‐−1.37)	(−12.04‐−1.43)	(−14.33‐3.80)	(−20.83‐−3.00)
Year after start* cohort 2	19.00**	19.00**	−3.54	−3.54
	(1.93‐36.07)	(1.96‐36.03)	(−32.73‐25.66)	(−32.16‐25.08)
Year after start* cohort 3	−42.38***	−42.38***	−7.41	−7.41
	(−49.88‐−34.89)	(−49.86‐−34.91)	(−20.23‐5.41)	(−19.97‐5.15)
Age		0.18**		2.62***
		(0.01‐0.35)		(2.34‐2.91)
Women		10.39***		−2.55
		(6.67‐14.10)		(−8.79‐3.69)
Observations	8026	8026	8026	8026
*R* ^2^	0.06	0.06	0.00	0.04

Note

Confidence interval in parentheses.

^a^ Days absent (baseline), cohort 1 used as reference.

*** *P* < .01;

** *P* < .05;

* *P* < .1.

The value of lost production naturally displayed a similar pattern (Table 1), with increased costs due to sick leave in the year following treatment initiation for cohorts 1 and 2, while this remained stable in cohort 3. The excess costs per person and year in the earlier treated cohorts is in the 7 000‐10 000 € range. No differences were observed for disability pension.

## 4 DISCUSSION

In this study, we observed a reduction in the number of days away from work due to sick leave in patients treated with IFN‐free treatment with the second DAA therapies compared to patients treated with ribavirin and/or peginterferon or with telaprevir or boceprevir added. To our knowledge, this is the first study of its kind.

The data from the National Social Insurance Agency do not contain enough details to allow us to determine the exact reason for these findings, but one possible explanation is the reduced burden of adverse events with the new drugs.^13^, ^14^ It is well known that peg‐IFN plus ribavirin had many adverse events. These were further increased with addition of the first‐generation protease inhibitors telaprevir causing frequent skin disorders and boceprevir causing in particular anaemia.^15^

There are some limitations to this analysis. We could only adjust our regression model based on a limited number of patient characteristics since no disease‐specific information was available from the national registries, and the quality register including more detailed information focuses on patients treated with IFN‐free treatment with the new DAAs. Although a similar proportion of patients underwent liver transplantation in the year following treatment initiation (0.7%, 0.5% and 0.5% in cohorts 1, 2 and 3), it is possible that the cohorts are different with regard to other factors. The first‐generation protease inhibitor telaprevir was mainly used in patients with advanced disease with genotype 1 hepatitis C virus infection, a majority with advanced fibrosis/cirrhosis. To manage costs, use of the second‐generation DAAs was initially restricted to patients with advanced fibrosis/cirrhosis—a restriction that was gradually lifted to finally include all patients as prices were negotiated down over time. Ribavirin + peg‐IFN cannot be used in patients with the most advanced cirrhosis including patients with Child C cirrhosis. Cohort 1 may therefore consist of somewhat less severe patients overall. It does, however, seem unlikely that this could explain the large difference in sick leave that was observed here since patients in cohort 3 initially mainly had liver disease with advanced fibrosis/cirrhosis including patients the most advanced cirrhosis which cannot be treated with peg‐IFN.^1^ Another data point supporting this argument is the fact that in cohort 3 where data on fibrosis stage are available, there is no impact of fibrosis stage as such on the difference in number of days of sick leave the year following treatment initiation compared to the previous year.

A difference‐in‐difference regression typically relies on an assumption about parallel trends, in this case that in the absence of intervention sick leave in the three cohorts would develop in a similar way. As we are in our estimation are relying on a single point of observation, it is not possible to formally test this assumption. With hepatitis C being a slowly progressing disease, the time frame of observation being short (one year pre and post intervention) and no other therapeutic innovations occurring during the time frame during which patients were included in the study, it seems likely that this assumptions should hold.

As our three cohorts are included at different points in time, changes in the regulations governing social benefits could potentially influence practices for physicians recommending sick leave. The regulations are constantly evolving, but during the time period under study we have not been able to identify any changes that would clearly influence this.^16^

The national social insurance agency only collects data on work absence that is covered by the national insurance. This period starts after 14 days, with the initial period being covered by the employer. This means that shorter spells of sick leave are omitted and that the total number of days of work absence is underestimated in all three groups.

In this study, lost production was valued according to the human‐capital approach where the lost work is valued according to the cost of employment which is a commonly applied method recommended by for instance the Dental and Pharmaceutical Benefits Agency in Sweden. An alternative valuation method, preferred by, for instance, the Dutch agency, is to use the friction cost method where costs are only accrued until a replacement can be found (during what is called the friction period) which requires more micro‐level labour market data. As the number of days away from work is fairly short overall (the 75th quartile was about 3 months), the two methods would give similar results in this case.

It can be noted that the estimate of work absenteeism likely does not capture the full effect of the new drugs as it only captures days off work during the first year after treatment initiation. With the new generation of drugs being more efficacious, there is also savings in the long run due to fewer patients progressing to more severe stages of disease (including cirrhosis). Measuring this would require a substantially longer follow‐up time, but there are recent modelling data from the United States that seem to support this notion.^17^

To conclude: our results highlight the importance of taking a broad societal perspective when discussing the cost and value of new therapies. The value of foregone production observed was between 7000 € and 10 000 € per treated patient, which is a significant part of the price of the new drugs at launch in Sweden. A broader cost perspective thus indicates a more pronounced cost‐benefit with these new DAAs than if only the perspective of the healthcare payer is taken in account.
